# Supplementary material for: Gut Microbiota and B Cell Receptor (BCR) Inhibitors for the Treatment of Chronic Lymphocytic Leukemia: Is Biodiversity Correlated with Clinical Response or Immune-Related Adverse Event Occurrence? A Cross-Sectional Study
Source: Microorganisms. 2023 May 17;11(5):1305. doi: 10.3390/microorganisms11051305 (PMC10223513; doi:10.3390/microorganisms11051305)
Supplement: Supplementary file 1 [file microorganisms-11-01305-s001.zip › microorganisms-2338405-supplementary.pdf]

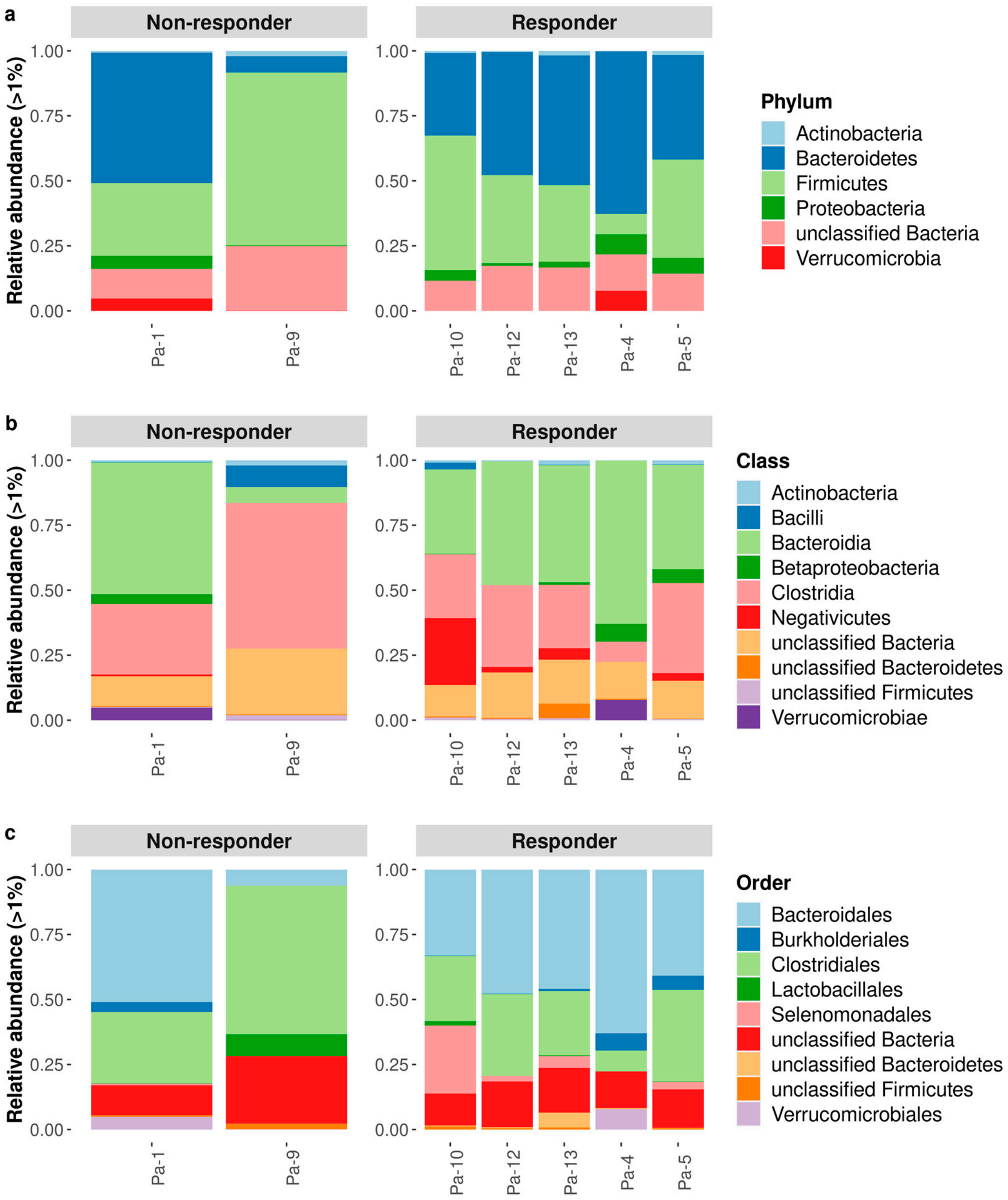

Supplementary Figure 1. Taxonomic composition and diversity of Fecal Bacterial Communities for each patient treated with Ibrutinib stratified for Responder and Non-responder group. Most represented phyla (a), class (b), order (c) identified in the study groups are shown with relative abundance. Only taxa whose relative abundance was > 1% in at least one group were included.

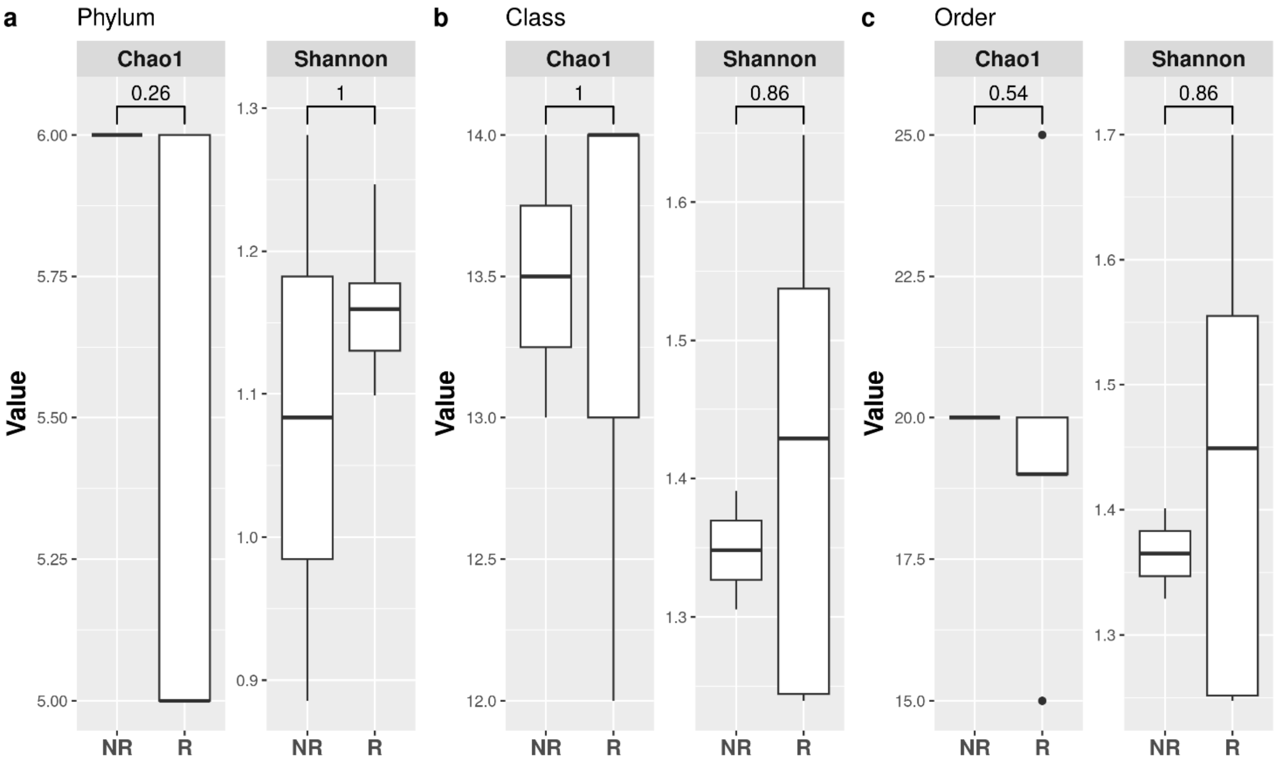

Supplementary Figure 2. α-diversity. Observed richness and Shannon indices are presented at the taxonomic level of phylum (a), class (b), order (c). Significant (** $p < 0.05$) comparisons between Responder (R) and Non-Responder (NR) patients treated with Ibrutinib are indicated in the boxplot.

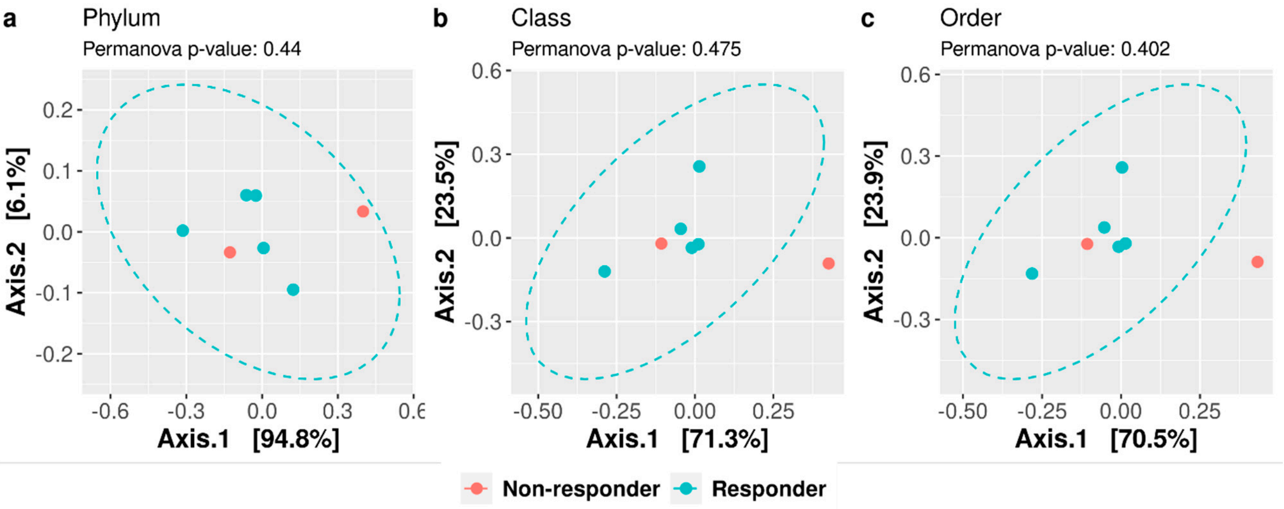

Supplementary Figure 3. Beta-diversity. The microbiota distances were evaluated through the Bray–Curtis dissimilarity matrix at the taxonomic level of phylum (a), class (b), and order (c) and visualized through Principal Coordinates Analysis (PCoA). Each point represents the microbiota composition of one sample and stratified by outcome (Responder and Nor-responder patients treated with Ibrutinib).
